# Transcription factor motif quality assessment requires systematic comparative analysis

**DOI:** 10.12688/f1000research.7408.2

**Published:** 2016-03-14

**Authors:** Caleb Kipkurui Kibet, Philip Machanick

**Affiliations:** 1 Department of Computer Science and Research Unit in Bioinformatics (RUBi), Rhodes University, Grahamstown, South Africa

**Keywords:** Motif assessment, Motif comparison, Motif scoring functions, ChIP-seq, Motif enrichment, Motif quality

## Abstract

Transcription factor (TF) binding site prediction remains a challenge in gene regulatory research due to degeneracy and potential variability in binding sites in the genome. Dozens of algorithms designed to learn binding models (motifs) have generated many motifs available in research papers with a subset making it to databases like JASPAR, UniPROBE and Transfac. The presence of many versions of motifs from the various databases for a single TF and the lack of a standardized assessment technique makes it difficult for biologists to make an appropriate choice of binding model and for algorithm developers to benchmark, test and improve on their models. In this study, we review and evaluate the approaches in use, highlight differences and demonstrate the difficulty of defining a standardized motif assessment approach. We review scoring functions, motif length, test data and the type of performance metrics used in prior studies as some of the factors that influence the outcome of a motif assessment. We show that the scoring functions and statistics used in motif assessment influence ranking of motifs in a TF-specific manner. We also show that TF binding specificity can vary by source of genomic binding data. We also demonstrate that information content of a motif is not in isolation a measure of motif quality but is influenced by TF binding behaviour. We conclude that there is a need for an easy-to-use tool that presents all available evidence for a comparative analysis.

## Background

**Figure 1.**
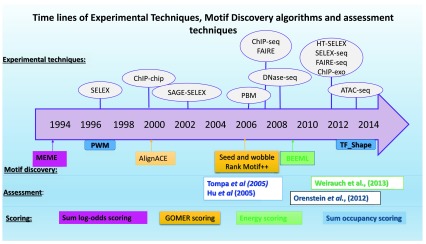
Evolution of motif scoring functions with experimental techniques and algorithms. Tompa *et al.* ^18^ and Hu *et al.* ^19^ assessed the motifs by binding site prediction while Orenstein *et al.* ^28^ and Weirauch *et al.* ^5^ used scoring. The scoring techniques are colour coded for the motif discovery or assessment where they were used.

Understanding gene regulation remains a long-standing problem in biological research. The main players, transcription factors (TFs), are proteins that bind to short and potentially degenerate sequence patterns (motifs) at gene regulatory sites to promote or repress expression of target genes. The search for a code to predict binding sites and model binding affinity of TFs has led to several experimental techniques and motif discovery algorithms being developed ( Figure 1).

A position weight matrix (PWM) is the common form of representing TF binding specificity. For a motif of length *L*, the corresponding PWM is a 4 × *L* matrix of probabilities of observing a base *b* (A, C, G or T) at position *i* through *L*. Other forms of representing TF binding specificity have been introduced ^1– 4^, but Weirauch *et al.* showed that a well-trained PWM performs comparably to some of the above well trained complex models ^5^. However, recent studies ^6– 8^ have reported significant improvement to the PWM by models that consider nucleotide inter-dependencies. The persistent popularity of PWM can be attributed to its simplicity and ease of use as well as the ease of visualizing a PWM using a sequence logo ^9^. Motifs can be found using a variety of methods including algorithms that do *de novo* motif discovery from sequences containing binding sites ^10– 12^ and *in vitro* methods such as protein binding microarrays (PBM) ^13^ and high-throughput systematic evolution of ligands by exponential enrichment (HT-SELEX) ^14^.

Initially, the low resolution of the available experimental techniques for TF binding specificity detection was a hindrance to the quality of binding models. However, next generation sequencing and techniques like chromatin immunoprecipitation (ChIP) followed by deep sequencing (ChIP-seq) ^15^ and exonuclease cleavage in ChIP-exo ^16^ that measure TF *in vivo* occupancy, have improved the resolution to single-nucleotide level. In addition to providing high resolution data for motif discovery, they are a useful resource to test the quality of the available motifs since they are TF specific. However, no benchmark capable of assessing the growing range of published motifs is available and quality measures are largely subjective ^17^.

Although it is possible that PWM models’ ability to describe TF binding may be getting saturated, the lack of a robust approach to test the quality of a model and maximize the best-performing ones may also be hampering improvement in performance. How are the algorithms being developed, tested and improved? Furthermore, the number of motif finding algorithms from dissimilar data sets and subsequently the number of motif models for a single TF generated, continue to increase. There are at least 44 PWM motif models available in 14 different databases for Hnf4a alone. How does the end-user decide which motif to use? In this study, we review and test the approaches used to evaluate TF binding models.

### Review of motif assessment approaches

The available motif assessment techniques can be divided into three categories: assess by binding site prediction, motif comparison or, by sequence scoring and classification.

### Binding site prediction

Early review and assessment of motif-finding algorithms tested tools on the ability to predict sites of motifs, known or inserted into the sequence. Tompa *et al.* tested motif discovery algorithms by their ability to predict sites of inserted motifs using statistical measures for site sensitivity and correlation coefficient ^18^. In this first comprehensive study, they found that a motif assessment problem is complex and admitted that inserting random motifs fails to capture the biological condition of TF binding. Later, Hu *et al.* ^19^ used real RegulonDB binding data in a large-scale analysis of five motif-finding algorithms. The tools available at that time performed poorly – “15–25% accuracy at the nucleotide level and 25–35% at the binding site level for sequences of 400 nt long” – largely due to the poor quality of RegulonDB annotations ^20^.

Sandve and colleagues ^21– 23^ tested motif discovery algorithms using sequences with real and inserted binding sites as benchmarks; from Transfac, and the third-order Markov model respectively. Quest and colleagues ^24^ developed the Motif Tool Assessment Platform (MTAP) as an automated test of motif discovery tools. However, this was computationally expensive and was made obsolete by new experimental data and algorithms.

The most comprehensive assessment based on binding site prediction so far has been by the Regulatory Sequence Analysis Tools (RSAT) consortium. In their ‘matrix quality’ script, they use theoretical – information content (IC) and *E*-values – and empirical scores computed by predicting binding sites in RegulonDB, ChIP-chip and ChIP-seq positive and negative control sequences ^20^.

Inadequate knowledge of TF binding sites has mainly hampered the ability to assess motifs and algorithms by binding site prediction. Predicting binding sites that are inserted or known in the sequences cannot accurately identify unknown true sites. Techniques that identify such sites may be penalized. Until TF binding sites are well annotated, this technique cannot be confidently utilized.

### Motif comparison

Novel motifs can be assessed by comparison to ‘reference motifs’ using the sum of square deviation, Euclidean distance and other statistics that measure divergence between two PWMs ^25, 26^. Thomas-Chollier *et al.* proposed a motif comparison approach for their RSAT algorithm where they combine multiple metrics, including Pearson’s correlation, width normalized correlation, logo dot product, correlation of IC, normalized Sandelin-Wasserman, sum of squared distances and normalized Euclidean similarity for each matrix pair ^27^. They then unified all of these scores to ranks whereby the mean of the ranks is considered the overall score.

Assessing motifs by comparison, as currently implemented, only tests similarity to the available motifs with little information on quality and ranks of the motifs. It assumes accuracy of ‘reference motifs’, with no way of assessing novel ones. In addition, the definition of ‘reference motifs’ remains largely subjective.

### Assessment by scoring

Motif assessment has since shifted towards scoring positive sequences known to contain binding sites and negative background sequences without binding sites, driven by high-throughput sequencing techniques ^5, 28– 30^. This avoids the need to identify binding sites *a priori* by focusing on the ability to classify the two sets of sequences. The differences in the assessments arise from the choice of sequences to use as positive and negative, the thresholds used to identify binding sites, the length of the sequences in both sets, the scoring function and the statistic used to quantify the performance of the tool.

For ChIP-seq data, the main difference is that the length of sequences (250bp ^28^; 600bp ^30^, 100bp ^5^ or 60bp ^31^) and the choice of negative sets (300bp downstream; ^28, 30^; random sequences, 5000bp from a transcription start site (TSS) or random genomic sequences ^5^, or flanking sequences ^31^) differ greatly in sequence scoring. In addition Agius *et al.* ^31^, test PWMs and support vector regression (SVR) models in the 36bp sliding window of the test sequences, a deviation from the rest of the techniques. All these differences, in addition to the scoring functions and statistics used, can lead to variations in the results of comparative analyses. Users and algorithm developers therefore have to frequently re-invent the wheel to test their tools.

Figure 1 shows the evolution of experimental motif discovery assessment techniques. We have not focused on the experimental techniques or motif discovery algorithms as excellent reviews are already available ^17, 32^. Rather, we focus on TF binding models represented as a PWM and aim to determine how the choice and length of benchmark sequences, scoring functions, and the statistics influence motif assessment. We hope that this study will highlight some of the pitfalls in the previous motif assessments and provide a starting point for a standard in motif assessment that will ensure comparability and reuse of results.

## Methods

### Data

Human uniform ChIP-seq data were downloaded from the ENCODE consortium ^33^ ( http://hgdownload.cse.ucsc.edu/goldenPath/hg19/encodeDCC/wgEncodeAwgTfbsUniform) (List of ENCODE data used available in Table_S3 ^66^). For each peak file, we used BEDTools v2.17.0 ^34^ to extract the 500 highest scored sequences (only peaks with no repeat masked sequences were used) of 50, 100, and 250bp centred on the ChIP-seq peaks as a positive set. Our choice for top 500 sequences was informed by our understanding of previous research ^35, 36^; using a TF-specific percentile of the available peaks did not make any significant difference (data not shown). A negative set of a similar number of sequences and length was extracted 500bp downstream from the highest coordinate (highest coordinate + 500) of the positive sequences.

When using PBM data to rank motifs, we mainly adopted the definition of positive and negative sets described by Chen *et al.* ^37^. A given motif is used to score a 36bp sequence for each spot using the different scoring functions. For this analysis, we only found nine TFs that had comparable data in ChIP-seq and PBM. These were: Egr1, Esrra, Gata3, Hnf4a, Mafk, Max, Myb, Pou2f2 and Tcf3. The data from Badis *et al*. ^38^ were downloaded from UNIPROBE database ^13^. A detailed Ipython notebook on this analysis can be found in https://github.com/kipkurui/Kibet-F1000Research.

**Figure 2.**
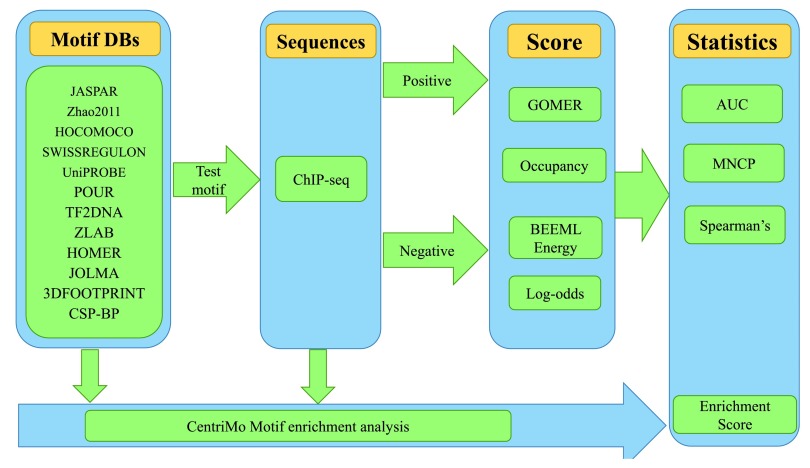
Methodology flow diagram. For a given transcription factor, all motifs available in various databases are extracted and used to score the given test sequences. The motifs are then ranked based on a given statistic.

We used motifs from a number of databases and publications listed in Table 1. The TFs used in this analysis were selected based on availability of ChIP-seq data with motifs in at least 10 motif databases. We converted these motifs from their various formats into MEME format and scored the positive and negative sequences with GOMER, occupancy, energy and log-odds scoring functions. We quantify how each motif performs using AUC, MNCP, Spearman’s and Pearson correlation ( Figure 2). This was implemented in a Python module which is available free from https://github.com/kipkurui/Kibet-F1000Research. This repository also contains raw data and Ipython notebooks that document how to reproduce the analysis we describe in this paper.

**Table 1.** Source of motifs used in the analysis. “Source” refers to the experimental technique used to generate the motifs while “mixed” motifs are generated using a variety of techniques. The specific motifs in MEME format used for this analysis are provided in the data repository ^66^.

Database	Source	Size	Reference
JASPAR	Mixed	127	39
UniPROBE	PBM	386	13
Jolma	HT-SELEX	843	14
Zhao	PBM-BEEML	419	40
POUR	ChIP-seq	292	41
HOCOMOCO	Mixed	426	42
SwissRegulon	Mixed	297	43
TF2DNA	3D Structures	1314	44
HOMER	ChIP-seq	264	45
Chen2008	ChIP-seq	12	35
3DFOOTPRINT	3D Structures	297	46
GUERTIN	ChIP-seq	609	47
CSP-BP	Mixed	734	48
ZLAB	ChIP-seq	409	36

### Scoring functions

When testing motifs by scoring ChIP-seq or PBM data, multiple scoring functions are available, which may affect the outcome. In the section that follows, we describe the scoring functions tested, as well as provide a review of how they have been previously applied.

### GOMER scoring

The GOMER scoring framework was introduced by Granek *et al.* ^49^ but adapted for PBM sequence scoring ^37, 38^. It seeks to compute the probability *g*( *S*,Θ) = *exp*( *f* ( *S*,Θ)) that a TF, given PWM Θ, will bind to at least one of the sub-sequences of *S*. This assumes that each site can be bound independently

$$g(S,\Theta )=1-\prod_{t=1}^{L-k}1-P(S_{t:t+k}|\Theta )\;(1)$$

where *L* is the length of sequence *S*, and *S*_*t*: *t*+ *k*_ is the sub-sequence of *S* from position *t* to *t*+ *k* inclusive. See Chen *et al.* ^37^ for more details.

### Occupancy score

The occupancy score calculates the occupancy of a PWM Θ for sub-sequence *S* ^*i*^ of length *k* as the product of the probabilities of each base in *S* ^*i*^ using equation 2.

$$f(S^{i},\Theta )=\prod_{j=1}^{k}\Theta_{j}[S_{j}^{i}].\;(2)$$

For a sequence *S* of length *L*, the sum of the occupancies of all sub-sequences *S* ^*i*^ (sum occupancy) ^28, 50^, the maximum score (maximum occupancy) ^30^, or the average occupancy (average motif affinity – AMA) have been used. Sum occupancy is defined in equation 3:

$$f(S,\Theta )=\sum_{t=0}^{L-k}\prod_{j=1}^{k}\Theta_{j}[S_{t+j}^{i}].\;(3)$$

### BEEML-PBM energy scoring

The energy scoring framework of binding energy estimation by maximum likelihood for protein binding microarrays (BEEML-PBM) ^4^ computes the logarithm of base frequencies with the idea that this is proportional to the energy contributions of the bases. The binding energy at each location is computed; the lower the binding energy, the higher the binding affinity. For each sequence, the sub-sequence with the lowest binding energy represents the score of the sequence. It has mainly been used to score PBM data ^5, 30^.

The probability that sub-sequence *S* ^*i*^ is bound is given by equation 4,

$$P(S^{i}\;is\;bound)=\frac{1}{1+e^{E(S^{i})-\mu }},\;(4)$$

where, for a sub-sequence *S* ^*i*^, *E*( *S* ^*i*^) is given equation 5,

$$E(S^{i})=\sum_{b=A}^{T}\sum_{t=1}^{L}\in (b,t)S^{i}(b,t),\;(5)$$

for binding site of length *L*, *∈*( *b*, *t*) is the energy contribution of base *b* while *S* ^*i*^( *b*, *t*) is an indicator function of site *t* within *S* ^*i*^(1 with base b, 0 otherwise).

### Log-odds scoring

In log-odds scoring, used by a majority of the MEME Suite tools ^51^, the score for a given site is the sum of the log-odds ratios of a PWM at the match site. For a sub-sequence *S* ^*i*^ of length *L* scored using PWM Θ, the log-odds score is given by equation 6,

$$LogOdds(S^{i},\Theta ,\;p)=\sum_{t=1}^{L}\sum_{b=A}^{T}S^{i}(b,t)log\frac{\Theta_{t,b}}{P_{b}}.\;(6)$$

where *p*_*b*_ the background probability (uniform background probability of 0.25 is used) and *S* ^*i*^( *b*, *t*) is an indicator function of site *t* as in equation 5.

The score for a given sequence can then be derived by summing individual scores or by finding the maximum score. Sum log-odds scoring has generally been used by MEME Suite tools while maximum log-odds scoring has also been used to compare motifs represented differently (PWM, *k*-mer and SVM models) against one another ^30, 31^. Each of these approaches has inherent advantages but may produce inconsistent results.

### Statistical measures of performance

With the scores of each motif for the sequences acquired, binding prediction can be evaluated by various statistics. The area under the receiver operating characteristic curve (AUC) ^52^ has been widely used, especially with the advent of PBM ^5, 28, 37^. In addition to popularizing AUC, Clarke *et al.* ^52^ also introduced a novel metric, mean normalized conditional probability (MNCP), for quantifying the correlation between DNA features and gene regulation. This statistic has been applied for motif assessment in GIMME motifs ^53^ and is said to be less affected by the presence of false positives compared with AUC since it places emphasis on true positives. MNCP is a rank-based statistic that determines if mean occurrence of a motif in test sequences is higher than the mean occurrence in a random set. Each set of sequences is ranked based on the mean occurrence, and the MNCP calculated by finding the mean of the normalized ratio of the two sets of ranks. We use MNCP to test how it contributes to better prediction in an effort to encourage its use.

Pearson and Spearman’s rank correlation are still widely used as a measure of motif performance. Spearman’s rank correlation has been used for PBM and ChIP-seq sequences ^28^ while Pearson’s correlation was used by Weirauch *et al.* ^5^. However, Weirauch *et al.* cautioned on the use of Spearman’s correlation for PBM data citing its inability to exclude low intensity probes. We wish to check the usefulness of correlation statistics in motif assessment.

In addition to comparing the scoring approaches, we use CentriMo version 4.10.0 in differential mode ^54^ – an option that tests differences in motif enrichment between two sequence sets – in a novel way for motif assessment. We set differential mode parameters for local rather than central enrichment of all the input motifs in the positive (primary) and negative (control) set, as described in the Data section, by using a very large threshold. The negative log of the *E*-value is used as the measure of a motif’s enrichment and rank. Motif enrichment analysis has previously been performed ^36^ using the FIMO algorithm ^55^ to scan for motif matches in sequences and calculate an enrichment value.

## Results

### Length of sequences has a little effect on motif performance

**Figure 3.**
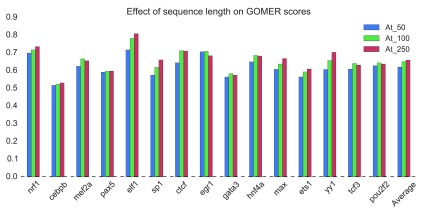
Effect of sequence length. Using all the motifs for each of the 15 TFs, we tested the effect of sequence length (50bp, 100bp and 250bp) using GOMER scoring on ChIP-seq data. For each TF, the mean of the AUC of the motifs is computed and the mean of all the 15 TFs computed to obtain the average. The motifs used in this analysis are available as supplementary data in the repository ^66^.

The size of the putative binding region – length of the sequences in each data set – is to some extent a proxy for how accurate the ChIP-seq experiment was. If the result was accurate a narrow region should contain the true site. For the three variants of sequence length, we did not observe a significant effect (p=0.113, for 50 and 100; p=0.0545, 50 and 250; p=0.678, 100 and 250bp – Wilcoxon rank-sum test) on the scoring of the sequences ( Figure 3). The scores assigned for each sequence length, however, seems to indicate how the TFs bind. Motifs with higher scores at lower sequence length (50 or 250bp) are generally enriched at the ChIP-seq peak, which is also a strong indicator of direct binding ^56^. This is consistent with a previous observation that a successful ChIP-seq experiment localizes binding within about 100bp of the true site ^57^. Others with significantly better AUC values at 250bp sequence length like Elf1 (p=0.017, Wilcoxon rank-sum test) and Sp1 (p=0.013, Wilcoxon rank-sum test) ^58^, are known to bind cooperatively.

### Tissue or cell line of the data could affect enrichment

**Figure 4.**
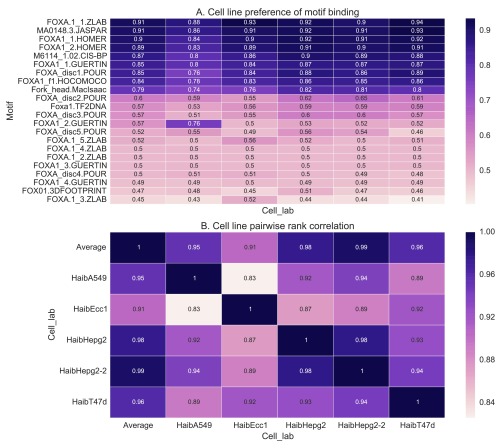
Cell line-specific binding. In **A**, we show how the ranks of the motifs can be influenced by the cell line used in the analysis. Foxa motifs are used to score each of the five cell lines using GOMER scoring and quantified with AUC values. Similar results are obtained with other scoring functions. In **B**, we show how the ranks assigned to the motifs are correlated among the cell lines.

Transcription factors bind to their possible sites in a sequence-specific manner. Some actually have alternative binding motifs depending on the tissue or cell line. Unless the purpose of motif assessment is to identify tissue-specific binding, if data is available from more than one cell line, an average of the scores should be used. For example, in Figure 4, we show that the rank correlation of the motif scores in different cell lines can be as low as 0.8 for GOMER scoring (or as low as 0.65 when energy scoring is used), and not 1 or very close to 1 as would be expected if the cell line had no effect. In addition, FOXA1_1.GUERTIN motif is differentially enriched only in the A549 cell line (although this could be an outlier).

In light of this possible effect, the results displayed throughout this paper are based on the mean score of all the available ChIP-seq data sets to avoid a bias towards cell line-specific motifs.

### How choice of negative (background) sequences affect motif ranking

**Figure 5.**
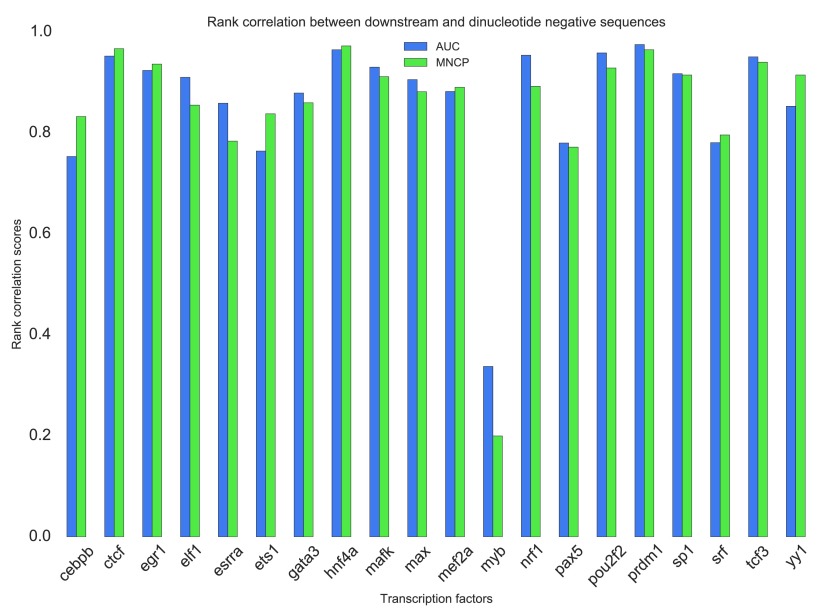
Influence of negative sequences on motif ranking. For each TF, the available motifs are used to score positive and two sets of negative sequence; downstream set and a dinucleotide shuffle of the positive set (see text for details). The figure displays a rank correlation of normalized MNCP and AUC scores from the two sets of negative sequences. Pearson and Spearman’s correlation do not require negative sequences therefore, they are not affected. We only show results based on GOMER scoring, but similar conclusions can be made from the other scoring functions.

In motif discovery, the choice of background sequences has significant effects on the motifs identified. We sought, therefore, to test whether motif scoring would be affected in a similar way. In addition to downstream sequences, we used a dinucleotide shuffled set from the positive sequences. The scores obtained using dinucleotide shuffled positive sequences were always lower than those for downstream sequences. We then computed and plotted the rank correlation of scores normalized by maximum score for each TF, from which we find that it affects the ranks of the motifs ( Figure 5) in a TF-specific manner. However, the scores from the two sets of negative sequences used are not significantly different (p=0.484, Wilcoxon rank-sum test). For Myb, the low correlation could be attributed to how it binds, indirectly ^59^.

### Effect of statistic on motif ranking

**Figure 6.**
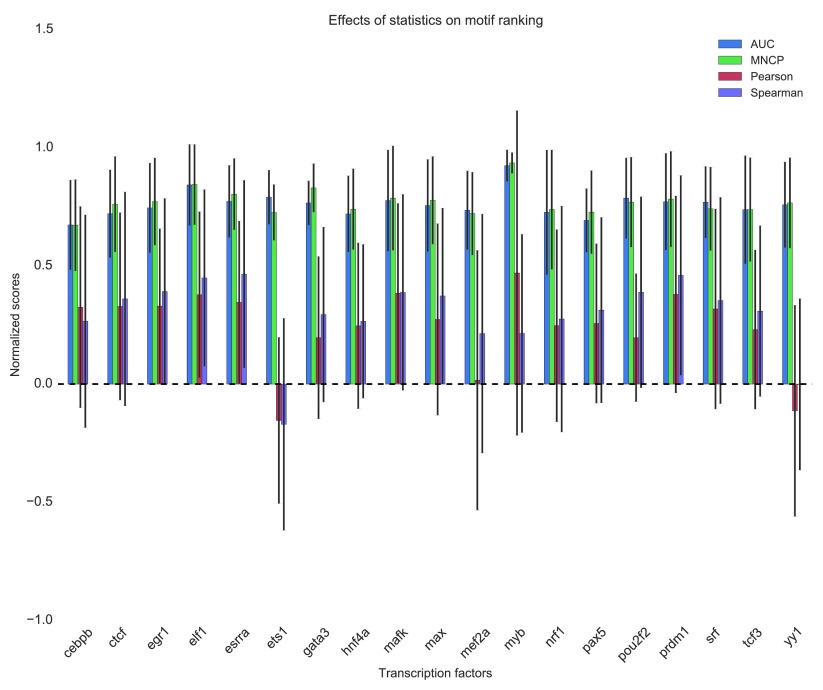
Effects of statistics on motif ranking. For each TF, the motifs are used to score sequences using GOMER scoring function and ranks determined by MNCP, AUC, Pearson and Spearman’s rank correlation. In this figure, we compute the mean normalized scores and compute the standard deviation for each TF, which is displayed as error bars.

The statistic used, whether it measures scores correlation or ability to classify the two sets of sequences, will definitely have an effect on how we interpret the results of the analysis. Generally, the motifs ranks based on AUC and MNCP statistics’ are not significantly different (p=0.52, Wilcoxon rank-sum test), but the ranks based on Pearson and Spearman’s differ significantly from MNCP or AUC scores (p=0.006 and 0.002 respectively, Wilcoxon rank-sum test). The large standard deviations of the correlation statistics’ scores, as shown by the error bars in the Figure 6, shows how unreliable the use of correlation statistics to rank the motifs can be. The correlation scores are also quite low.

### Effect of scoring function is transcription factor specific

**Figure 7.**
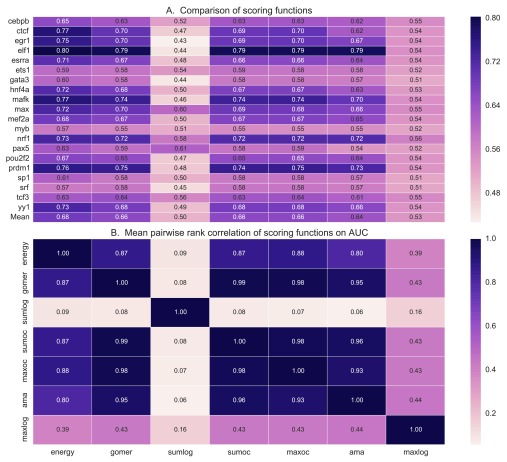
Effect of scoring function on motif ranking using AUC statistic. **A**. For each TF, the mean AUC score is computed for each of the scoring functions used. In **B**, we show how the ranks assigned to various motifs for a given TF by each scoring function are correlated. It displays the pairwise rank correlation for all TFs in **A**. *Sumlog*: Sum log-odds function, *Sumoc*: sum occupancy score and *Maxoc*: maximum occupancy.

**Figure 8.**
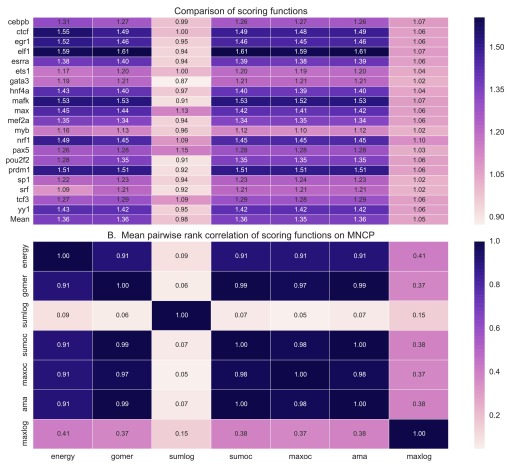
Effect of scoring function on motif ranking based on MNCP statistic. See caption in Figure 7 for details.

We tested the ability of PWM models to discriminate positive (top 500 peaks of width 100bp centred on the peak) and negative (500 peaks 100bp wide located 500bp downstream from the positive) sequence sets using five scoring functions. Maximum and sum log-odds scoring had low discriminative power for most of the motifs when AUC ( Figure 7) and MNCP ( Figure 8) statistical measures are used. However, sum log-odds scoring had some good performance (over 0.55 AUC scores) for some TF motifs like Max, Nrf1, Tcf3 and Pax5.

There is no significant difference in performance when GOMER, energy or occupancy scores (sum, maximum and AMA) are used for scoring ( Figure 7B) with AUC statistic (see Table_S1 for details of statistical significance). Also, we did not observe any significant difference (p=0.85, Wilcoxon rank-sum test) between sum occupancy and maximum ( Table 2), contrary to a claim by Orenstein *et al.* ^28^. When using MNCP, there is a higher rank correlation among the scores assigned by the different scoring functions except log-odds scoring ( Figure 8B). When using AUC or MNCP statistic, Ctcf, Egr1 and Hnf4a score significantly higher in energy while other TFs like Pou2f2 and Esrra, the preference is reversed. These observations are reflective of the inherent features of the scoring functions or the statistics used.

**Table 2.** Mean scores and standard deviation (SD) of AUC and MNCP for scoring functions. For each transcription factor, the median and mean for AUC or MNCP are computed for all the available motifs. *Sumlog*: Sum log-odds function, *Sumoc*: sum occupancy and *Maxoc*: maximum occupancy.

Statistic	Energy	GOMER	Sumlog	Sumoc	Maxoc
Mean AUC	0.68	0.66	0.5	0.66	0.66
Median AUC	0.7	0.67	0.48	0.64	0.64
AUC SD	0.15	0.15	0.11	0.15	0.15
Mean MNCP	1.36	1.36	0.98	1.36	1.35
MNCP SD	0.27	0.32	0.14	0.32	0.31

### Motif length and information content

**Figure 9.**
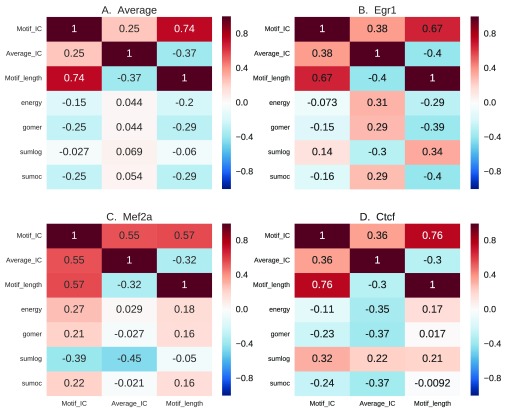
Effect of motif length and IC on scoring functions. In this figure, we show the correlation of motif length, full length information content (IC) and the assessment scores, to determine how performance of scoring functions are influenced by motif characteristics. For each motif, the information content is calculated based on information theory for the whole length and also normalized for length. The results for average motif affinity (AMA) and maximum occupancy are similar to sum occupancy, and are not included.

Motif length has little bearing on the quality of motif, independent of other factors. However, specific motifs with very high IC such as those from POUR have a lower performance ( Figure 9). In the same light, those motifs with low IC also have a lower performance *in vivo*.

The heat map in Figure 9 shows how the motif scores from the four discriminative functions correlate with motif length, full-length IC and average IC. The examples have no consistent correlation between the IC and the scores ( Figure 9A). However, there is a negative correlation between both the total IC and motif length, and the scores except for sum log-odds scoring, which has no significant correlation (p=0.34, correlation p-value). This shows that motif length, rather than the IC of the motifs, negatively influences the scores assigned by these functions. This is not a general rule. Some TFs exemplify a different scenario. For example, Egr1 ( Figure 9B) has a positive correlation between IC and scores and a negative correlation with motif length (except for sum log-odds scoring), showing that it has a highly specific binding site ^60^. Mef2a, on the other hand, has a positive correlation between motif length and scores showing preference for longer low information motifs ( Figure 9C). This could also reflect variability in binding sites. Ctcf has the highest negative correlation for average IC, with a neutral to positive correlation for motif length ( Figure 9D), which may indicate preference for longer low IC motifs.

### Comparison of motif databases

We have shown that the effect of scoring algorithms is TF-specific. We also test to see how, overall, the different databases (DBs) are ranked against each other. For TFs with more than one motif in a given DB, we use the best performing one to represent the DB. We also use motif enrichment-based assessment using CentriMo version 4.10.0 to provide more evidence to scoring based techniques’ results. Motif enrichment analysis compares how various motifs in foreground sequences are enriched compared with background sequences. In comparing how two or more motifs for the same TF perform, the level of enrichment of the motif in sequences known to contain possible binding sites of the TF compared to some background should provide a measure of the quality of the motif.

**Figure 10.**
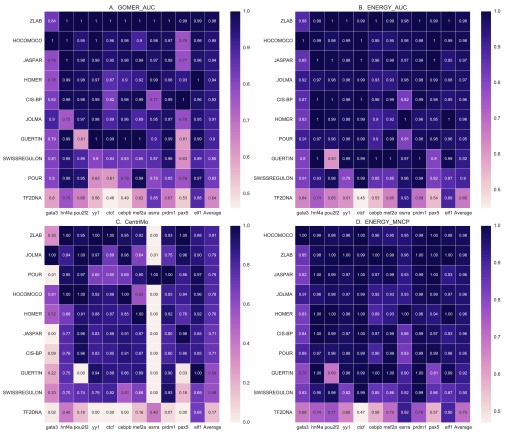
Ranking of motif databases. We compare the motif databases by using the best ranking for each motif using GOMER and energy AUC and MNCP values, and CentriMo enrichment values. For each scoring function, the scores for each TF are normalized by dividing each value with the maximum, which are then averaged to rank the different databases.

Figure 10 provides a summary of ranking of the various databases for the given TFs. We observed that the performance of a majority of the motif databases did not differ much, except for TF2DNA motifs, but the ranking or the performance of individual motifs differs. This further supports the observation of TF-specific performance of scoring function, algorithms and DBs. It also shows that no single database currently outperforms the others for all TFs. There is agreement in ranking of the best (ZLAB and HOCOMOCO) and worst performing (TF2DNA and SWISSREGULON) DBs. We observe that, compared with GOMER ( Figure 10A), the ranks for most DBs remain the same when using energy ( Figure 10B) except for POUR and JOLMA. This shows that motifs from these DBs, or at least the best performing ones, may be favoured by energy scoring. It is also noteworthy that POUR and GUERTIN DB motifs, discovered and tested on ENCODE ChIP-seq data, generally perform poorly.

### Effect of PBM data on motif assessment

**Figure S1.**
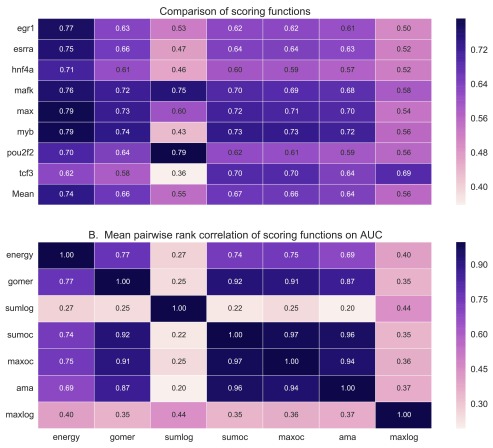
Effect of scoring function on motif ranking using AUC statistic for PBM data. **A**. For each transcription factor (TF), the mean AUC score is used to represent it for each scoring functions used. In **B**, we show how the ranks assigned to various motifs for a given TF by each scoring function are correlated. It displays the pairwise rank correlation for all TFs in **A**. *Sumlog*: Sum log-odds function, *Sumoc*: sum occupancy score and *Maxoc*: maximum occupancy.

**Figure S2.**
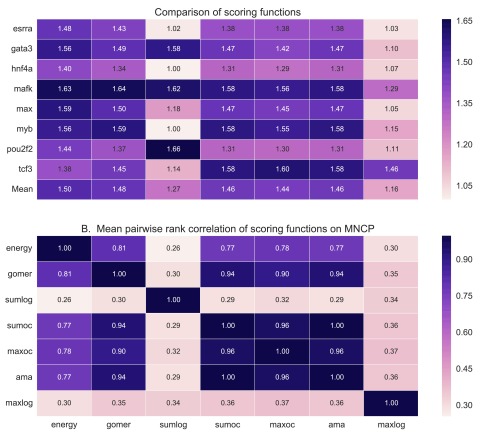
Effect of scoring function on motif ranking based on MNCP statistic in PBM data. See caption in Figure S1 for details.

**Figure S3.**
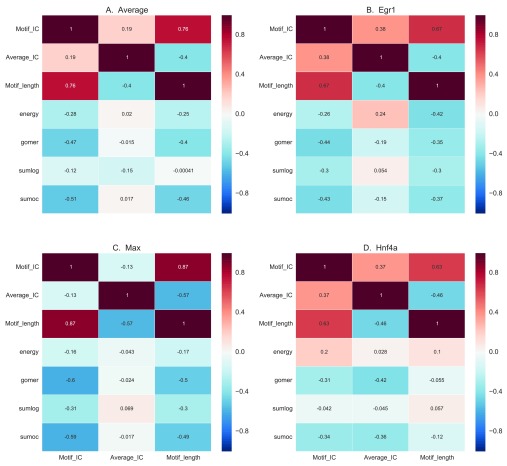
Effect of motif length and IC on scoring functions using PBM data. In this figure, we show the correlation of motif length, full length information content (IC) and the assessment scores, to determine how performance of scoring functions are influenced by motif characteristics. For each motif, the information content is calculated based on information theory for the whole length and also normalized for length. The results for average motif affinity (AMA) and maximum occupancy are similar to sum occupancy, and are not included.

**Figure S4.**
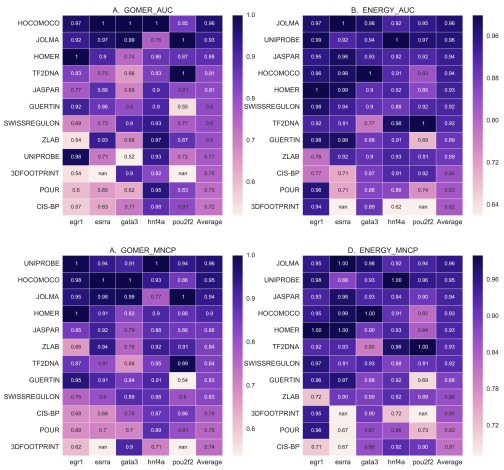
Ranking of motif databases when based on PBM data. We compare the motif databases by using the best ranking for each motif using GOMER and energy AUC and MNCP values, and CentriMo enrichment values. For each scoring function, the scores for each TF are normalized by dividing each value with the maximum, which are then averaged to rank the different databases.

To test whether the conclusions of the paper are only linked to ChIP-seq data, we re-ran the whole analysis using PBM data from the UniPROBE database ^13^. It is important to note that we only found 9 TFs that had PBM data from the set used in ChIP-seq analysis. Since this may bias the comparison, we compared with a similar set in ChIP-seq and found the observations below were not affected by the difference in number of TFs used. These observations include:

1. A much higher energy score in PBM ( Figure S1 and Figure S2) compared with ChIP-seq ( Figure 7 and Figure 8). We also observe a much lower correlation between the energy and the occupancy scores.
2. A stronger negative correlation between the occupancy scores and motif length -0.47 compared with -0.28 of energy scoring ( Figure S3), an observation not made when using ChIP-seq data ( Figure 9). This may actually explain observation 1.
3. Motifs generated using the PBM technique perform best when using occupancy scores with MNCP or energy scores with AUC or MNCP, except when occupancy scoring and AUC are used ( Figure S4). Poor ranking of UniPROBE PBM-derived motifs by GOMER-AUC may be linked to the fact that they penalize long motifs – UniPROBE motifs are know to be long (mostly over 14bp).
4. Energy scoring with either MNCP or AUC, or occupancy scoring with MNCP display similar behaviour: a preference for specific motifs, which may be longer or have a higher IC. This supported by the high negative correlation between motif length and occupancy scores with AUC ( Figure S3).
5. TF2DNA motifs perform better when PBM data is used ( Figure S4A) compared with ChIP-seq data ( Figure 10A), and especially so when GOMER scoring is used together with AUC statistic. It is not immediately clear what the cause of the difference of performance of TF2DNA motifs in PBM and ChIP-seq data is, but the short length (7bp) of TF2DNA motifs and the fact that they were generated *in vitro* could provide some explanation given that PBM data are generated in 8-mers and PBM is also an *in vitro* technique.

## Discussion

We have described a comparative analysis on the effect of scoring functions, ChIP-seq test data processing and statistics on motif assessment. We chose to focus on TF binding models represented as a PWM, since it is most commonly used. The review reveals the complexity of the motif assessment problem, showing no appropriate solution is available so far. The available techniques focus on testing motif algorithms or the experimental techniques used, but little has been done to compare the available motifs for a given TF. There is a need for a tool, accessible and easy to use by end-users, to aid in choosing motifs.

The use of 100 or 250bp sequence length provides necessary discrimination for the TFs tested ( Figure 3). The performance was also found to be TF specific, an observation that could reflect inherent binding behaviour; direct, indirect or cooperative binding of the TF. This supports the observation that direct binding can be inferred from ChIP-seq peaks ^56^. We also confirm that 100bp provides acceptable specificity in motif assessment given that most TF binding sites are less than 30bp ^57^.

Since TF binding is cell line specific ^61^, users should be aware of bias caused by use of one cell line in an assessment. We observe that the use of more than one cell line reduces the bias towards cell line specific motifs ( Figure 4).

The MNCP rank-order metric is similar to AUC but derived by plotting true positive hits against all sequences’ scores. This places emphasis on true positives and therefore is less affected by false positives. Most of the observations from the PBM-based analysis support the conclusion that energy scoring prefers specific motifs (long or with a high IC). We also observe an agreement when energy scoring is used with AUC and MNCP, or occupancy scoring. In MNCP, the preference for specific motifs is expected because the MNCP score provides a rank-based measure of the ratio of mean occurrence of a motif in test sequences and a random set. These observations are not conclusive and further research may be required. Although there is no clear winner among the scoring function, occupancy-based (GOMER, AMA, sum and max) and energy scoring functions are preferred. We recommend, based on the presented evidence, using occupancy scoring with the MNCP statistic or energy scoring with normal AUC or the MNCP statistic.

There is no significant correlation (p=0.513, correlation p-value) between the IC and the motif scores ( Figure 9). This compares with the conflicting observations that the best-quality motifs may have low IC motifs ^5^, or high IC motifs ^62^. Weirauch *et al.* did not normalize for motif length, which results in high IC motifs that are generally longer but not necessarily more specific ^5^. A shorter motif with higher IC per position will be more specific but have lower total IC. We argue that the effect of IC on motif quality is dependent on the TF binding behaviour. TFs with short and specific binding sites will favour high IC while those with long and variable binding sites will be more accurately modelled with low IC. Furthermore, it has been shown the low IC flanking motif sites contribute to specificity of binding *in vivo* ^62^. We have also shown that the techniques used in motif assessment have a direct effect on motif discovery. We observe how motifs generated from similar data using the same techniques could have highly variable performance in POUR, ZLAB and GUERTIN motifs ( Figure 10). This difference in quality can be explained by motif discovery algorithms used, data processing as well as the assessment techniques used in each motif discovery pipeline. POUR motifs are learned from full-length sequences of the top 250 peaks using five motif finding algorithms (MEME, MDscan, Trawler, AlignAce and Weeder) ^41^, the ZLAB group used 100bp of the top 500 sequences centred on the ChIP-seq peaks using MEME-ChIP ^63^, while GUERTIN reports the top 5 motifs for each technique generated using MEME. For quality assessment, POUR ^41^ used a TFM-PVALUE ^64^ to match motifs against the testing ChIP-seq data set and the most enriched motifs against a background composed of intergenic non-repetitive regions. ZLAB group used FIMO ^55^, which uses a log likelihood score for motif scanning.

The worst performing motifs, from TF2DNA, are generated from 3D models of TF from experimental or homology-modelled PDB files. When generating these models, they determined the accuracy of the models based on similarity to UniPROBE and JASPAR motifs at a given threshold. They claimed their technique successfully identifies true motifs 41–81% of the time depending on the quality of templates used in modelling 3D structures. We speculate that part of the reason for this low performance could be use of motif comparison against ‘reference motifs’ as a measure of motif quality, in addition to being *in vitro* derived. Better performance of TF2DNA motifs in PBM data ( Figure S4) further supports this observation. Although JASPAR and UniPROBE are widely used, reliance on reference motifs is problematic, especially with the advent of motif databases like HOCOMOCO and CIS-BP that have motifs with better prediction quality. As a general principle, it is not reasonable to use historical data as a benchmark for assessing potentially better new methods.

We also show that the choice of data used in motif assessment has a direct effect on the ranks of the motifs. It goes without saying that PBM-derived motifs will perform better when tested with PBM data or for ChIP-seq based motifs tested on ChIP-seq data. The main criteria for choosing the test data should be based on the intended use of the motifs. In addition, we confirm the effect of negative (background) sequences in motif assessment, an effect well known in motif discovery.

We have confirmed that motif assessment has transcription-specific variability, an observation previously made ^65^. Assessments should be less focused on how a particular motif database or algorithm performs but on how different motifs, for a particular TF, perform compared to the other potential motifs. For the end user, no single database can provide the sole measure of quality of new data. This raises the need for collation of the different motifs tested using a variety of motif assessments to provide information to the end user on their ranks.

## Conclusions

We have demonstrated that the scoring techniques used in motif assessment influence ranking of motifs in a TF-specific manner. Motif assessments and tools developed to date have focused on comparing algorithms, experimental techniques or databases. This does not help the user choose which motif to use for a given TF. Some TFs reviewed here have at least 44 PWM motifs available, raising the need for a tool that can be utilized to rank these motifs. We have also shown that data processing as well as motif assessment can have a significant influence on the quality of motifs derived. Therefore, the choice of an assessment approach should be given as much thought as that of the motif discovery algorithm to use. We have also shown the effect of IC on motif quality is influenced by TF binding behaviour.

In short, a single measure of motif quality is likely to remain elusive, pointing to the need for tools and methods for comparative analysis using multiple methods. Lessons learned from this analysis will be useful in a number of ways. Firstly, we are working on a web-based application that can allow users to compare motifs available in different databases for a specific TF. Secondly, we intend to extend the motif by comparison approach to avoid ‘reference motifs’ bias. Thirdly, we have shown the effect of motif scoring on motif discovery. We intend to use the robust motif assessment techniques we introduce to improve motif finding.

## Data and software availability

Data, software, supplementary files and documentation for ‘Transcription factor motif quality assessment requires systematic comparative analysis’ are available from Github: https://github.com/kipkurui/Kibet-F1000Research.

Archived files at the time of publication are available from Zenodo: doi: 10.5281/zenodo.46440 ^66^

## Supplementary information

This section provides supporting figures for the paper.
