# Supplementary material for: Transcription factor motif quality assessment requires systematic comparative analysis
Source: F1000Res. 2016 Mar 14;4:ISCB Comm J-1429. Originally published 2015 Dec 11. [Version 2] doi: 10.12688/f1000research.7408.2 (PMC4821295; doi:10.12688/f1000research.7408.2)
Supplement: Peer review history

# Peer review: Referee response for version 2

**DOI:** 10.5256/f1000research.8713.r13169

**Published:** 2016-04-01

**Authors:** Trevor W. Siggers

**Affiliations:** 1 Department of Biology, Boston University, Boston, MA, USA

I accept this revised version of the manuscript. I am satisfied with the updates provided by the authors.

I have read this submission. I believe that I have an appropriate level of expertise to confirm that it is of an acceptable scientific standard.

# Peer review: Referee response for version 2

**DOI:** 10.5256/f1000research.8713.r12883

**Published:** 2016-03-31

**Authors:** Jan Grau

**Affiliations:** 1 Institute of Computer Science, Martin Luther University of Halle-Wittenberg, Halle, Germany

I am happy to state that the authors thoroughly addressed most of my concerns regarding the previous version of the manuscript.

However, I still have a few minor comments, which the authors might consider, as listed in the following.

1. In the introduction, the authors state that Chip-seq has improved "resolution to single-nucleotide level", whereas at the beginning of the Results section, they state that "a successful ChIP-seq experiment localizes binding within about 100bp of the true site", which still is quite accurate but not exactly single-nucleotide accuracy. Please clarify.
2. In section "Background", sub-section "Motif comparison", the authors list as possible measures of motif divergence "sum of square deviation, Euclidean distance". As the former is just the square of the latter, I would suggest to give another example (for instance, correlation-based meaures) in the introduction of that sub-section.
3. In section "Methods", sub-section "Data", the authors now describe the selection of negative sequences, which were "extracted 500bp downstream from the highest coordinate".
  a) However, as ChIP-seq peaks lack an orientation, I wonder which direction "downstream" refers to. Does this mean that the authors considered the forward strand of the available genomic sequence, or do they also take, e.g., the orientation of closely located genes into account?
  b) In the response, the authors explain that "our focus was to get negative sequences [...] which maintains the nucleotide composition". As I mentioned in my previous review, sequences located 500bp downstream of a transcription factor binding site might already be located in coding sequence, which affects nucleotide composition. Could the authors please comment on this issue?
4. In section "Methods", sub-section "Data", the authors explain that they "only found nine TFs that had comparable data in ChIP-seq and PWM". However, there are additional, similar data sets for at least Foxo1, Zfx, Tbx5, and Nr5a2 available (see Grau *et al*., 2013 for details). Please clarify.
5. In equation 1, parentheses might help to spot the argument of the product.
6. In equation 4, the parameter μ is not explained.
7. In Figure 3, the authors discuss the influence of sequence length on AUC. However, I would consider the influence of sequence length on the ranking of motifs more relevant for the topic of the manuscript.
8. In section "Results", sub-section "Effect of PBM data on motif assessment", 5th item of the list, the authors state that "PBM data are generated in 8-mers". As far as I know, PBMs are typically designed such that they cover all 10-mers (see, e.g., http://the_brain.bwh.harvard.edu/pbm.html). In addition, the authors might clarify which data they used for the assessment (the probe sequences and intensities, I assume?).

I have read this submission. I believe that I have an appropriate level of expertise to confirm that it is of an acceptable scientific standard.

## References

[1] GrauJPoschSGrosseIKeilwagenJ: A general approach for discriminative de novo motif discovery from high-throughput data. *Nucleic Acids Res*.2013;41(21) : 10.1093/nar/gkt831 e197 10.1093/nar/gkt831 24057214PMC3834837

# Peer review: Referee response for version 1

**DOI:** 10.5256/f1000research.7983.r11721

**Published:** 2016-01-15

**Authors:** Jan Grau

**Affiliations:** 1 Institute of Computer Science, Martin Luther University of Halle-Wittenberg, Halle, Germany

The manuscript "Transcription factor motif quality assessment requires systematic comparative analysis" by Kibet and Machanick addresses the assessment of transcription factor binding motifs. This question is especially important for selecting appropriate motifs for computational predictions given the large number of different motifs for the same transcription factor available from databases. Kibet and Machanick specifically consider different measures for motif scoring and assessment, and investigate different factors that might influence the assessment and, hence, the chosen motif.

The topic is of great relevance in any research dealing with sequence motifs and a systematic analysis of the factors influencing their assessment may help to develop a standardized framework for motif assessment.

However, I have several reservations regarding the current version of the manuscript as outlined below.

As a general comment (that does not necessarily require a response by the authors), I found it slightly disappointing that the present manuscript does pose many important questions and potential obstacles in motif assessment, but does not provide a solution, be it guidelines for reasonable motif assessment or be it even a platform for performing such analyses.

DATA:

1. I wonder why the authors decided to only consider ChIP-seq data but no *in-vitro *data (PBMs or SELEX). While *in-vivo* binding may of greater relevance for many applications, problems like cell type-specificity of motifs (also addressed by the authors) would have a minor influence. In addition, competitive or interaction effects with other transcription factors might be ruled out. Finally, some of the motif sources considered derive their PWMs from *in-vitro* data. In summary, an analysis also using *in-vitro* data might affect the conclusions of the paper.

2. For all ChIP-seq data sets under consideration, the authors extract (only) the top 500 highest scoring sequences for the assessment. This may have largely differing effects for different transcription factors, where, for instance, one transcription factor might have several hundred ChIP-seq positive regions, whereas another transcription factor might have tens of thousands of ChIP-seq positive regions. Hence, in one case also lowly occupied sequences are collected whereas in the other case, the positive data set may only contain the most stringent binding sequences. This may affect all downstream analyses and, for instance, could be one of the reasons why the authors observe transcription factor-specific effects for some factors. Hence, I would strongly suggest to conduct the analysis with a transcription factor-specific selection of sequences (where the simplest idea might be to use just a percentile).

3. a) The authors state that they construct a "similar" negative set. Here, the authors should clearly define what "similar" means, how sequences are selected, and how many negative sequences are in the set. 

b) In addition, the specific selection of negative sequences described by the authors (500bp "downstream", where "downstream" is also unclear as ChIP-seq regions lack an orientation), might introduce a specific bias, because under the assumption that transcription factor binding sites are often located close to the transcription start, which might mean that the negative sequences may already be coding and, hence, per se different from promoter sequences.

c) Finally, from my experience, the choice of the negative data set strongly affects the performance assessment of motifs. Hence, the authors might consider to test an additional set of negative sequences (e.g., di-nucleotide shuffled positive sequences) in their analysis.

METHODS:

4. The "Methods" section, especially formulas needs substantial revision:

a) In general, notation should be harmonized between the different formulas. For instance, the sequence S appears with different indexes with different meanings; the indicator function is denoted by S_i(b,m) in eqn. (5) and by I(S_{i,b}) in equation (6).

In addition:

b) In eqn. (1), parentheses are missing around (1 - P(...)). In addition, the notation S_{t+1:t+k}^{i} is not explained

c) In eqn. (2), it is unclear if i and [S_{t=i}] are indexes or if this should denote a product of \theta, i and S_{t=i} (which I consider unlikely). In addition, the variable t (in the index) is neither bound nor explained.

d) In eqn. (3), the upper limit of the sum is |s|, where it should be |S|, I assume. In addition, there seems to be something missing (a \theta?) in the product.

e) Before eqn. (5), the authors refer to T as the length of the sequence. However, considering the formula, the length should be L, and the first sum from A to T refers to the alphabet. In addition, eqn. (6) again denotes the sum over the alphabet differently.

f) In eqn. (5), the text refers to sequence S but the formula to sequence S_i

g) In eq. (6), the variable P_b is not defined (the authors later only refer to p, which might have the same meaning). In addition, the authors to not explain, which background distribution they use in the assessment, which will be relevant, e.g., for the results presented in Fig. 6.

5. The energy scoring framework (eqn. 4 and 5) and the LogOdds scoring framework are formally defined only for sub-sequences and it remains unclear how these are applied to longer sequences from ChIP-seq. Are those subjected to the occupancy definitions (maximum and/or average) as well?

6. LogOdds scoring is referred to as "Log likelihood scoring" in the section's title (page 6, left column), which is not fully correct.

7. On page 6, right column, second paragraph, the authors state that they "wish to check the usefulness of correlation in motif assessment" (which I would find interesting), but I did not find any results regarding correlation as performance measure in the results.

RESULTS:

8. In several cases, the figure captions are too minimalistic to understand the contents of the figure. I would suggest to spend a few more sentences in the captions to explain the main idea of each figure. In addition, not all of the abbreviations are explained in the caption of Fig. 6.

9. On page 6, penultimate paragraph, the authors state that "the Foxa motif from the POUR data set is significantly differentially enriched only in the A549 cell line", which I could not read from Fig. 4. Please clarify.

10. On page 8, right column, the authors state that "MNCP prefers specific motifs, which will have more true positives". Could the authors elaborate on these findings and also possibly give an (mathematical) explanation?

11. In Fig. 6, the authors show AUC values for different motifs and scoring functions. 

a) First, it remains unclear which data sets have been used in this analysis for the different transcription factors. Is it just the average over all motifs and data sets for each factor? 

b) Second, I did, unfortunately, not get the general idea of this analysis. If I understood it correctly, the main question of this manuscript is to study the effects of different factors on motif assessment with the goal of selecting the most appropriate motif for a given transcription factor. However, here it seems to be that exactly this information is averaged out. Wouldn't be the more interesting question how the scoring functions affect the ranking (by AUC) of the different motifs for each transcription factor?

12. On page 10, left column, the authors state that they "did not observe any significant difference (p=0.85, Wilcoxon rank-sum test) between sum occupancy and maximum (Table 3)". However, I did not find maximum occupancy listed in Tab. 3.

13. On page 11, right column, the authors state that Egr1 has strong positive correlation between IC and scores. However, I found this correlation not too strong for Average_IC and in most cases not even positive for Motif_IC.

14. a) In remains unclear, what exactly is shown in Fig. 8. I speculate that the authors computed the correlation of AUC values, IC and motif length for different data sets and motifs? Or is it really correlation between occupancy/energy and IC/length? 

b) In addition, most of the entries of the heatmaps show correlations between the occupancies/energy, which, however, is not discussed. If correlation between occupancies/energy is not of interest, the authors might consider omitting all but the first three rows of the heatmaps. 

c) Further, I wonder why the correlation between identical entries (e.g., Motif_IC with Motif_IC) is not equal to 1 in panel A.

15. On page 12, second paragraph, the authors explain that they used the best performing motif to represent each database. However, this will introduce a bias towards larger databases, because these may contain a larger number of motifs for a transcription factor and, hence, are allowed to try a larger number of options, of which the best is chosen. I would suggest to use another, less biased statistic (e.g., the median) instead/in addition.

16. The authors also use CentriMo scoring for comparing databases, which they did not consider before, and I wonder what is the reasoning behind using CentriMo in this case (and not before).

17. In Figure 9, panel C, the authors rank the databases by average CentriMo score, while the magnitude of scores differs greatly between transcription factors and, hence, is dominated by data sets with large scores (e.g., cebp). I would suggest to level the influence of transcription factors, for instance by dividing the values in each column by their maximum value before averaging.

18. On page 12/14, the author state that "This supports our view that use of motif comparison against ‘reference motifs’ as a measure of motif quality is not reliable". While I agree with the general conclusion of the authors, I do not see why the performance of TF2DNA supports this conclusion. If only 41-81% of the TF2DNA motifs are correct (according to comparison against reference motifs), I would have expected a lower performance compared to the other databases.

OTHER/MINOR:

19. In section "Background", second paragraph, the authors refer to Weirauch *et al*., stating that a well-trained PWM performs comparably to more complex models. While this correctly describes the finding of Weirauch *et al*., several publication in the meantime came to different conclusions (e.g., Kulakovskiy *et al*., 2013 ^1^; Mathelier & Wasserman, 2013 ^2^; Mordelet *et al*., 2013 ^3^; Keilwagen & Grau, 2015 ^4^). Hence, the authors might consider to make this statement more balanced.

20. In section "Background", fourth paragraph, the authors state that "the quality of models derived has not improved in a comparative manner". I am not fully sure if I understand the statement correctly, but if the authors mean that the experimental techniques have improved, but the motifs did not (or much less), I would challenge this statement and at least encourage the authors to provide a reference.

21. The authors should provide a list (or a link to a list in their repository) of the specific ENCODE data sets used in the analysis.

22. Table 1: Chen2008 should be ChIP-seq data.

23. As performance measures, the authors consider the area under the ROC curve and MNCP. While the former might be familiar for most working in the field, the authors might consider to give a short formal definition of MNCP. In addition, the area under the precision-recall curve might be another useful measure for imbalanced data sets. [However, depending on the construction of the negative data set, the test data might even be balanced.]

24. Typos & Grammar:

- Page 4, second paragraph: "Sandev" should be "Sandve"

- Page 4, 5th paragraph: "Sandelinâ-Wasserman" should be "Sandelin-Wasserman"

I have read this submission. I believe that I have an appropriate level of expertise to confirm that it is of an acceptable scientific standard, however I have significant reservations, as outlined above.

## References

[1] KulakovskiyILevitskyVOshchepkovDBryzgalovLVorontsovIMakeevV: From binding motifs in ChIP-Seq data to improved models of transcription factor binding sites. *J Bioinform Comput Biol*.2013;11(1) : 10.1142/S0219720013400040 1340004 10.1142/S0219720013400040 23427986

[2] MathelierAWassermanWW: The next generation of transcription factor binding site prediction. *PLoS Comput Biol*.2013;9(9) : 10.1371/journal.pcbi.1003214 e1003214 10.1371/journal.pcbi.1003214 24039567PMC3764009

[3] MordeletFHortonJHarteminkAJEngelhardtBEGordânR: Stability selection for regression-based models of transcription factor-DNA binding specificity. *Bioinformatics*.2013;29(13) : 10.1093/bioinformatics/btt221 i117-25 10.1093/bioinformatics/btt221 23812975PMC3694650

[4] KeilwagenJGrauJ: Varying levels of complexity in transcription factor binding motifs. *Nucleic Acids Res*.2015;43(18) : 10.1093/nar/gkv577 e119 10.1093/nar/gkv577 26116565PMC4605289

# Response

**Published:** 2016-02-10

**Authors:** Caleb Kipkurui

**Affiliations:** Rhodes University, South Africa

Thank you very much for your insightful comments and recommendations. They have helped us improve the paper.

The main aim of this paper is to identify the weaknesses and potential pitfalls in the current techniques used in motif assessment. As part of our conclusions, we state that we will use the findings of this paper to develop a motif assessment platform to address the questions and the gaps. That work is almost done and should be available by March 2016, and is therefore out of the scope of this paper.

1. We focused on ChIP-seq data in assessment since we believe that, for most cases, the final utility of the motifs learned is predicting *in vivo* binding of the motifs. That said, we agree that data used in testing the motifs does have an effect on the ranking of the motifs. This is an observation we confirm with our re-analysis using PBM data. We have included a section on how assessment in PBM and ChIP-seq are influenced differently (Effect of PBM data on motif assessment). 

2. Our choice for top 500 sequences was informed by our understanding of previous research. However we did not make it clear that such prior work supports this point, and we have now cited a reference. As advised, we decided to test if this would affect the results from this analysis. The ChIP-seq peaks we use have a median of 14000 peaks, the highest having 92,258 peaks and a minimum of 101 peaks. Where the number of the available peaks was less than 500, we used all the peaks. Given the median number of peaks, we found 5% of the peaks to be appropriate and we used  this when 5% the of the total was more than 500, else we used top 500 peaks (or all of them, for data sets smaller than this). We also tested for 10% of the peaks. In all this, we found that the size of the peaks used had no significant effect on the results obtained. We, therefore, eliminate that as being one of the reasons for cell line specific binding.  This may, however, have an effect on cell line specific ranking behaviour even if we did not observe that in our examples, given that the number of peaks differs for a given TF in different cell lines.  We will definitely consider this suggestion when developing our tool to avoid any potential bias caused by this.

3. a) The manuscript has been updated. By similar we mean in size and sequence length.

b) In our analysis, downstream is based on the coordinates of the peaks. We extracted sequences located 500bp from the highest coordinate (highest coordinate + 500). Our focus was to get negative sequences which are not expected to contain binding sites for the given TF but which maintains the nucleotide composition. That distance, whether it falls in a promoter region or not, should be appropriate in for our specific analysis ***.***

c) On other negative sequences that can be chosen, we agree that this can have some influence in the analysis. The scores obtained when a negative set generated using  dinucleotide shuffled positive sequences were always lower than those from downstream sequences. However, the ranking of the motifs did not change in any significant manner. We expect random negative sets to have a significant influence on the ranks of the motifs and their probable difference in GC content from the binding region makes their appropriateness questionable. 

4. The notations used in the formulas have been updated for uniformity. Thank you!

5. For energy scoring, the subsequence with the lowest energy is used to represent the sequence while for logOdds scoring, the score can either be obtained by getting the sum or the maximum score for all the sub-sequences. Clarified in the paper, thank you.

6. Corrections have been made in response to 6, 8 and 9.

7. We have updated the Figure 5 to include details on the usefulness of correlation statistics. We find them to produce significantly different ranks from MNCP or AUC or even between Pearson and Spearman correlations.

8. Done

9. Done

10. We have updated the paper in to add a line giving more information about MNCP. Simply put, the MNCP is a rank-based statistic that determines if the mean occurrence of a motif in test sequences is higher that the mean occurrence in a random set. Each set of sequences is ranked based on the mean occurrence, and the MNCP is calculated by finding the mean of the normalized ratio of the two ranks. 

11. We have updated Figure 6 (now Figure 7) to address the comments. Our earlier figure actually averaged the information on the effect of scoring functions on the ranks of the motifs. We have updated by using the rank correlation of the motifs for various TFs to show how it affects ranking.**

12. Table 3 (now Table 2) has been updated to include maximum occupancy.

13. On Egr1 motifs correlation of motif IC and scores, we have updated the statement to be in accordance with the data. 

14. In Figure 8 (now Figure 9) we have updated the figure to only retain relevant columns. We have also corrected the error that led to identical entries’ correlation being more than 1. 

15. On why we chose to use the best motif's score to represent a database, we argue that since the focus of this analysis is to test our ability to choose for the best motif, irrespective of the database, we find using the best motif score to represent the DB to be sufficient. Besides, using median will still lead to biased results since DBs with many motifs of low quality and a few of high quality will be poorly ranked. 

16. We only introduce CentriMo at a later stage of our analysis as an alternative method of scoring techniques to motif assessment. The focus of the paper was to systematically assess the factors that do influence motif assessment, so we wanted to maintain that focus. 

17. We have taken your suggestion on Figure 9 (now 10) to normalize the scores. Thank you.

18. On the performance of TF2DNA, we agree that the low performance would be expected. We also believe that a different approach to motif assessment during motif discovery may have produced better motifs. In addition, testing using PBM data produced a much better performance. This may be a consequence of the motifs being short and only generated using *in vitro* methods.

19. The background section has been updated to include to making the observations balanced and including recent citations.

20. We accept that our statement on the lack of significant improvement of the motifs may have been misleading and unsupported. We have updated it to reflect current evidence. 

21. A list of the ENCODE data we use has been added to the repository

22. The source of Chen2008 updated to ChIP-seq from PBM in table 1. Thank you

23. A definition of MNCP has been added to the paper. We had previously tested area under a precision-recall curve and found it to produce similar results to AUC. 

24. Typos corrected

Once again, thank you.

# Peer review: Referee response for version 1

**DOI:** 10.5256/f1000research.7983.r11604

**Published:** 2016-01-05

**Authors:** Trevor W. Siggers

**Affiliations:** 1 Department of Biology, Boston University, Boston, MA, USA

The manuscript by Kibet *et al. *“Transcription factor motif quality assessment requires systematic comparative analysis” addresses an important issue in the field of regulatory genomics, namely how we analyze motif enrichment in genomic datasets. The authors have addressed this issue in a systematic way by compiling many datasets and versions of motifs, and analyzing the impact of different scoring methods.

This type of meta-analysis will be of interest to a wide audience. However, the current manuscript needs considerable revision. In particular, the connections between the data presented and the conclusions reached need to be strengthened and clarified. Furthermore, a lot more clarification about what is being shown in the figures is needed to properly evaluate the conclusions. Below I have outlined specific examples through to Figure 8.  The figure legends could definitely use more detail to help clarify what is being shown, and there needs to be more explicit and careful connection between the data and the conclusions (see examples below). I think that the type of analyses contained in this manuscript will be of interest to a wide audience; however, the manuscript needs to be substantially revised.

**Table 1. **Is Chen2008 databases, Reference 39, really PBM data?

**Methods/Data.**  For each peak file, the 500 highest scored sequences were identified “after eliminating repeat masked sites”.  It is a little unclear what this statement means. Does that mean that no peak was selected if there was any repeat masked seqeuence within the 50, 100 and 250bp windows? Or was the repeat masked sequence just masked and the genomic window extended to attain the 50, 100 and 250 bp cutoffs? Also, for the negative set, does ‘similar’ mean length-matched? It was exactly clear how this negative set was constructed.

**Figure 3 /results.** It was not clear why only a subset of 15 of the Encode ChIP-seq datasets were used and shown here, and how many datasets were used in the ‘Average’?  Also, the figure caption notes that ‘all the motifs for the 15 TFs’ were used, but it’s not clear how many that was and whether the reported AUC values were averages over their individual AUC values? I little more clarification would be helpful.

**Page 6.**  The authors write, “Unless the interest is tissue-specific binding, if more than one set of data is available, an average should be used”. Used for what? For motif discovery? 

**Figure 4.**  Why was ‘energy scoring’ used for this enrichment analysis, while GOMER scoring was used in Figure 3? Are the results dependent on these scoring differences? If not, then for consistency sake, it would be helpful to limit the enrichment analyses to a single scoring scheme.

**Page 6/Figure 4.** The authors conclude, “the Foxa motif from the POUR data set is significantly differentially enriched only in the A549 cell line and not so much in the other cell lines”.  I have no idea to what the authors are referring here, and this is the only conclusion from Figure 4. There are 5 different FOXA_discX.POUR motifs, all of which seem to score about the same on the different ChIP datasets. There is a FOXA1_2.GUERTIN that seems to be quite different, but this seems like an outlier within the dataset. I do not see how the data supports the contention that there are specific FOXA motifs that are better suited to particular ChIP datasets, it seems that for the most part they agree. Much more clarity is needed here.  

**Page 8 / Figure 5.**

“However, in some situations like Hnf4a and Ctcf, they are not (Figure 5)”. I only see Ctcf data represented in Figure 5, this should be clarified.

“The motifs ranked higher only by MNCP are generally long or with high IC (Table 2)”.  It would be much easier to see this if they were indicated somehow in Figure 5, perhaps with arrows are stars or something. Second, these conclusions don’t seem to follow from the data at all. The CTCF_disc1.POUR seems also to score high with Energy_AUC, so it’s not clear that the MNCP is the only factor of relevance here.  The CTCF.1_5.ZLAB seems to be most affected by the Energy vs GOMER scoring, and not the MNCP approach.  Even if these issues were resolved, it is impossible to know whether these motifs are ‘generally long or with high IC’ from Table 2, because the other motifs aren’t shown. It would be much clearer if the mean and variance of the length & IC for all motifs were also provided for context, or even better correlate the relative score AUC to MNCP differences by length or IC, to truly see if a trend exists.

**Figure 6.** 

Please clarify in the figure legend whether these values are for averages over multiple ChIP datasets (as was discussed above), and if so how these averages are determined.

“Maximum and sum log-odds scoring had low discriminative power for most of the motifs when all three statistical measures are used (Figure 6)”. What are the three statistical measures you’re referring to, and where’s the data? I only see data for AUC. Please clarify.

**Table 3. **Please be explicit in the figure legend about what the ‘Mean’ and ‘Median’ refer to (i.e., mean and median AUC values calculated over X single motif analyses described in Figure 6)

**Figure 7/ page 10.** “The variation in the scores is particularly reduced when MNCP statistic is used (Figure 7)”.  How am I supposed to see this? What is a significant difference in MNCP and how does it compare to a difference in AUC.  Based on the coloring scheme presented the results in Figure 6 and Figure 7 look very similar- it is not clear at all that there is any qualitative difference between these two figures except for the different measures used (i.e., an appropriate normalization might make them near equivalent).

**Figure 8.**  It is not clear (nor mentioned) what is being shown in this figure. I assume – but I could be wrong – that we’re looking at AUC values for each factor (i.e., Mef2a etc) averaged over some ChIP-seq datasets, but how are these being compared to each other? Further, how is Motif_IC which is a function just of the PWM being compared to a scoring function. I can’t speak to the conclusions being reached as I don’t currently know what data is being shown. Much more clarification is warranted in the text and figure caption.

I have read this submission. I believe that I have an appropriate level of expertise to confirm that it is of an acceptable scientific standard, however I have significant reservations, as outlined above.

# Response

**Published:** 2016-02-10

**Authors:** Caleb Kipkurui

**Affiliations:** Rhodes University, South Africa

Thank you very much for taking the time to review our paper and provide recommendations.Your comments have been very helpful in improving the paper.

**Table 1**

Corrected

**Methods/Data**

On repeat masked sequences, we have updated the paper to clarify the we did not include any sequences in test or negative sequences that contained masked positions. By a similar set of negative sequences, we mean matched in length and number of sequences. 

**Figure 3/Results**

The figure caption is updated to clarify. The number of the TFs used was decided based on the availability of ChIP-seq data as well as having motifs in more than 10 of the databases used. A list of the motifs used is provided in the data repository as well as the specific ChIP-seq data used. See also Methods / Data paragraph 3. 

**Page 6:**

On cell line specific binding, an average of the scores of all the available cell lines should be used in motif assessment.  We have updated the statement for clarity.

**Figure 4**

We have changed to using results from GOMER scoring since they are similar; the effect described is only pronounced in Energy scoring. 

**Page 6/Figure 4**

We had incorrectly mentioned the wrong motif to be significantly enriched. We have corrected this and also provided further evidence to the effect that the cell line used in the assessment does actually have an effect on the ranking of the motifs. The conclusions remain valid. 

**Page 8/Figure 5 (now Figure 6).**

We acknowledge that the figure we had used did not present the intended information correctly. We changed the figure to present the general information on the effect of statistics on the ranking of the motifs. We observe that, when normalized, the MNCP and AUC scores do not differ, except for slight difference in some TFs like Hnf4a, Ctcf, Gata3. However, the Pearson and Spearman's correlation scores vary greatly. The plot of the standard deviation of scores as represented by error bars in Figure 6 demonstrates why we consider correlation scores to be reliable than the other scores. We have added clarification of this point to the paper. Thank you again for pointing out the problem.

**Figure 6 (now Figure 7)**

The caption has been updated for clarity. 

**Table 3 (Now Table 2)**

Clarified

**Figure 7 (now Figure 8)/page 10**

The figure has been updated to include information on correlation statistics. 

**Figure 8 (now Figure 9)**

Our apologies for the lack of detail. The figure caption has been updated for clarity. We correlate the scores for the various motifs (for each scoring function) to the length and information content of the motifs to determine whether the scores obtained are in any way influenced by the motif characteristics.
